# Can clinical observation differentiate individuals with and without scapular dyskinesis?

**DOI:** 10.1590/bjpt-rbf.2014.0025

**Published:** 2014

**Authors:** Newton Y. Miachiro, Paula M. F. Camarini, Helga T. Tucci, Kevin J. McQuade, Anamaria S. Oliveira

**Affiliations:** 1 Ribeirao Preto Medical School (FMRP), Universidade de São Paulo (USP), Ribeirão Preto, SP, Brasil; 2 Department of Human Movement Sciences, Universidade Federal de São Paulo (UNIFESP), Santos, SP, Brasil; 3 University of Washington, Division of Physical Therapy, School of Medicine (UW), Seattle, WA, United States of America

**Keywords:** biomechanics, shoulder, health evaluation, validation studies, rehabilitation

## Abstract

**Background:**

Altered scapular rotation and position have been named scapular dyskinesis. Visual dynamic assessment could be applied to classify this alteration based on the clinical observation of the winging of the inferior medial scapular border (Type I) or of the prominence of the entire medial border (Type II), or by the excessive superior translation of the scapula (Type III).

**Objective:**

The aim of this study was to determine if there were differences in scapular rotations (Type I and II) and position (Type III) between a group of subjects with scapular dyskinesis, diagnosed by the clinical observation of an expert physical therapist, using a group of healthy individuals (Type IV).

**Method:**

Twenty-six asymptomatic subjects volunteered for this study. After a fatigue protocol for the periscapular muscles, the dynamic scapular dyskinesis tests were conducted to visually classify each scapula into one of the four categories (Type IV dyskinesis-free). The kinematic variables studied were the differences between the maximum rotational dysfunctions and the minimum value that represented both normal function and a small dysfunctional movement.

**Results:**

Only scapular anterior tilt was significantly greater in the type I dyskinesis group (clinical observation of the posterior projection of the inferior angle of the scapula) when compared to the scapular dyskinesis-free group (p=0.037 scapular and p=0.001 sagittal plane).

**Conclusions:**

Clinical observation was considered appropriate only in the diagnoses of dyskinesis type I. Considering the lower prevalence and sample sizes for types II and III, further studies are necessary to validate the clinical observation as a tool to diagnose scapular dyskinesis.

## Introduction

Previous studies have defined the normal movement of the scapula during arm elevation as a synchronized sequence of superior rotation and posterior tilt^1^ ^-^ ^3^ with a variable amount of medial-lateral rotation^3^. Variation of this dynamic pattern and of the resting position of the scapula on the thorax has been called 'scapular dyskinesis'^4^.

The identification of the bony prominences on the scapula at rest and during the dynamics of the scapulothoracic rhythm is challenging for clinicians because of the layers of tissue - in addition to the skin, the subcutaneous fat and muscles that cover the bone^4^. Additionally, the visual inspection is uniplanar by nature, while the dynamic dysfunction of the scapulothoracic rhythm is three-dimensional (3D)^4^.

Kibler et al.^5^'s classification, derived from observing the dynamics of the scapular dyskinesis combined with the rest position of the scapula, resulted in three dysfunctional patterns^5^. Type I was characterized, at rest, by the posteriorly displaced or winging of the inferior medial scapular border, and during arm elevation, by the posterior winging of the inferior angle of the scapula. Type II was characterized by the projection of the entire medial border of the scapula at rest and in motion. Finally, type III was characterized by excessive superior translation, with elevation and some anterior displacement of the superior border of the scapula on the thorax. A symmetrical pattern and the normal scapulothoracic rhythm were classified as type IV.

Other patterns of dynamic classification for scapular dyskinesis have been recently proposed in the literature, with acceptable values of intra- and inter-rater reliability^6^ ^,^ ^7^. Uhl et al.^6^ proposed a nominal dichotomous classification in which "NO" represents the absence of dyskinesis, and "YES" a positive diagnosis, if the clinician could observe any of the three dysfunctional patterns previously described by Kibler et al.^5^. McClure et al.^7^ classified the presence of dyskinesis as "subtle" or as an "obvious abnormality", according to the subjective perception of the examiner on the dysrhythmia or on the scapular winging. While a dorsal displacement was determined by winging of the medial border and / or the inferior angle of the scapula posteriorly, the dysrhythmia included premature or excessive elevation or protraction, irregularity (non-smooth) or judder (stuttering) during arm elevation or lowering, or a fast scapular inferior rotation during arm lowering. The criteria idealized by McClure et at.7, therefore, eliminated the necessity of a judgment based on observation of the specific direction of rotations or mal-positioning of the scapula. McClure patterns are qualitative (subtle or obvious), which created extra difficulty for its validation.

Although these patterns of classification have shown values ranging from poor to moderate^5^ ^,^ ^6^ or moderate^7^ for the inter-rater agreement, and moderate for the intra-rater agreement^5^, the only attempt to validate, by comparing three-dimensional (3D) kinematic data with the four diagnostic patterns described, showed that the asymmetries were common in both symptomatic and asymptomatic volunteers^6^.

Scapular dyskinesis has been associated with a variety of shoulder pain conditions^3^ ^,^ ^8^. However, the sample sizes and the designs of the studies were insufficient to support the idea that the dysfunctions of the scapulothoracic rhythm were risk factors for the development or perpetuation of the symptoms. Recently, a study^9^ conducted on 62 athletes involved in overhead sports, with 31subjects classified with obvious dyskinesis and 31subjects classified as normal^7^, showed a scapulothoracic rhythm pattern with decreased upward rotation and elevation and increased clavicular protraction for the diagnostic of the dysfunction. However, the observational diagnosis of scapular dyskinesis was not correlated to with the presence of shoulder pain^9^.

The three-dimensional measurements of the shoulder joint complex yields a relatively precise description of the position and orientation of the joint^1^ ^,^ ^10^. However, the cost of the equipment and procedures involved in data collection hampers the development of epidemiological (transverse or longitudinal) studies based on these data. Thus, the validation of the clinical observation method in the diagnosis of scapular dyskinesis is a challenge but would contribute to any investigations of how this dysfunction influenced pain in the shoulder joint complex.

The present study aimed to compare the differences in the rotations (types I and II) and position (type III) of the scapula between two groups, those with and those without scapular dyskinesis, that were classified by an experienced physical therapist through clinical observation, in accordance with the criteria proposed by Kibler et al.^5^. Our hypothesis was that individuals classified with scapular dyskinesis, when compared to the group without dyskinesis, would present increased anterior tilt of the scapula when clinically classified as type I, increased medial rotation as type II and increased superior translation as type III, as outlined by the protocol proposed for this study.

## Method

### Participants

A total of 26 volunteer subjects, 15 men and 11 women, with a mean age of 22.8 (±3.4) years and body mass index of 21.9 (±2.8) kg/m², were evaluated. According to the history and physical assessment, the subjects in the study had full and painless glenohumeral range of motion, absence of cervical spine and upper limb dysfunctions and would be classified as "sedentary"^11^ or those who "practice irregular physical activity"^11^ that did not involve the upper limbs. Subjects who reported any systemic conditions, history of trauma, surgery to the trunk, neck or upper limb, deformities of the spine or positive signs of orthopedic and neurological tests of the shoulder (except dyskinesis), elbow, wrist, hand and cervical spine, were excluded from the study.

All subjects were informed of the objectives and procedures involved in the study and signed a formal informed consent approved by the Ethics Research Committee of the Hospital das Clínicas, Ribeirão Preto Medical School, University of São Paulo (USP-HCFMRP), Ribeirão Preto, SP, Brazil, protocol No. 13032/2011.

### Procedures

The subjects were initially informed of the procedures and were able to familiarize themselves with the movement of elevating and lowering the arm in the sagittal and scapular planes. To assist the subjects in keeping the movement of the arms in the scapular and sagittal planes, a guide made of two PVC tubes were placed vertically in front of the volunteers at a distance that would orient the plane of arm elevation without the subjects touching the guide. The tubes were placed at an angle of 90º and 40º anterior to the frontal plane to guide arm elevation and lowering in the sagittal and scapular planes, respectively.

The 3SPACE Liberty (Polhemus. Inc, Colchester, VT) apparatus was integrated with (Innovative Sports Programs, Chicago, IL) *The Motion Monitor* software for collection of the 3D kinematics at a sampling frequency of 120 Hz. The electromagnetic sensors were firmly attached using adhesive tape and Velcron(r). Five acquisition sensors were positioned on either side of the body of the subjects as follows: two on the flat surface of the acromion of each scapula, to prevent movement produced by the adjacent soft tissue; two positioned on the humerus, just below the insertion of the deltoid muscle; and one placed on the sternum, just below the jugular notch. Each sensor provided information on position and angular orientation of the body segments with 0.08 cm RMS accuracy for position and 0.15º RMS accuracy for orientation, according to the manufacturer. The validity of the 3D kinematic measurements with the electromagnetic system was previously tested^12^ and the reliability of the intra-section was determined prior to the beginning of the study and was considered excellent^13^ (95% confidence intervals of the values of the intraclass correlation coefficient_2,k_ ranging from 0.94-1.00) for assessing the rotations and translations of the scapula on the dominant and non-dominant limbs.

The digitalization of the anatomical landmarks for defining local coordinates of the scapula, humerus and thorax was performed in accordance with the recommendations from the International Society of Biomechanics (ISB) for the upper extremity^14^. The axes YX'Z'' represented the movements of medial/lateral and superior/inferior rotations, and anterior/posterior tilt of the scapula.

**Figure 1:**
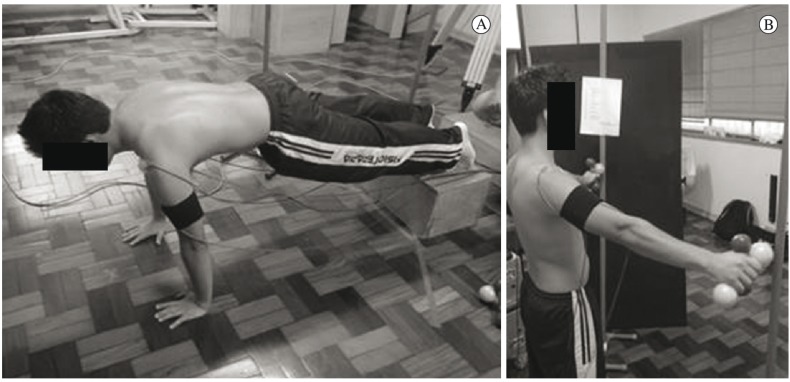
Fatigue exercise protocol of the periscapular muscles performed before the clinical observation of the scapulothoracic rhythm: (A) Position 1: isometric contractions in the push-up plus (maximal scapular protraction) position, and (B) Position 2: resisted standing scaption.

Before the clinical assessment and collection of the 3D kinematics, the subjects carried out a protocol performed to fatigue (fatigue protocol) of the peripheral scapular muscles, in which the subjects received a verbal encouragement. The fatigue protocol consisted of two parts: 1) an isometric contraction in the push-up plus position^15^ ^,^ ^16^ (maximum scapular protaction) (Figure 1A) holded as long as the volunteer stand, and 2) repetitions of bilateral, active, weighted arm elevation (Figure 1B). Load for active arm elevation was defined by body weigth as in McClure et al.^7^. The protocol was interrupted only after the subject reported an inability to continue the exercise combined with the observation of the following compensations: decreased scapular protraction or loss of trunk alignment at the *push up plus* position (for position 1) and inability to elevate the arm with full elbow extension without changing the plane of motion or during shoulder abduction with load (for position 2). The protocol was performed before the assessments because it had previously been demonstrated that peripheral muscle fatigue could alter scapulothoracic rhythm^17^. In addition, other functional tests also contribute to muscle fatigue and could exacerbate movement disorders^18^ ^,^ ^19^. The subjects completed the Borg Scale of Perceived Exertion^20^ before and immediately after the fatigue protocol.

**Figure 2:**
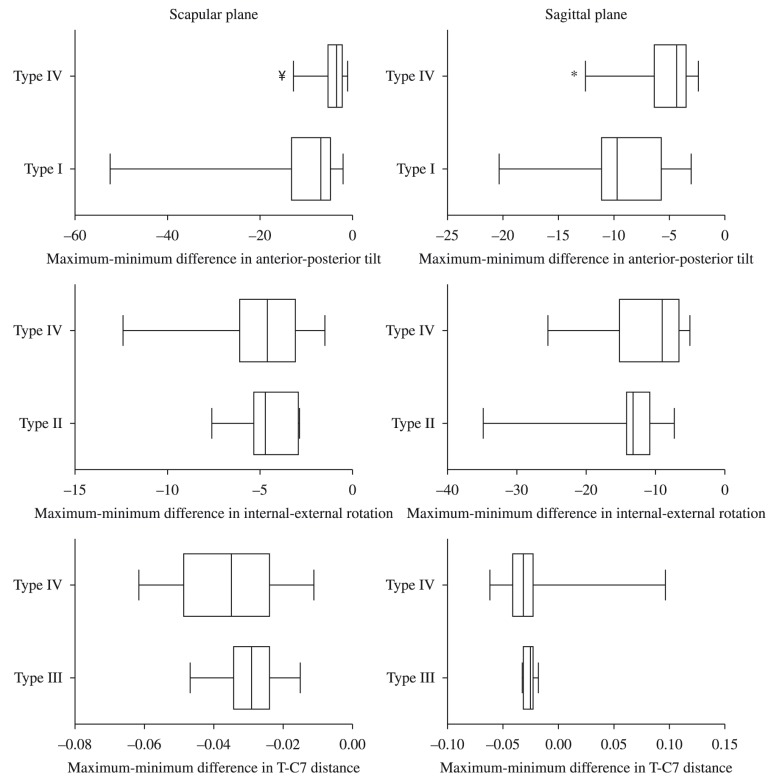
Box-plot showing the first to third quartile (vertical lines), the median, (vertical line inside the box) and the maximum and minimum values (horizontal lines) of the differences between the maximum and minimum anterior-posterior tilt (n=18), anterior-posterior internal-external rotation (n=9), superior translation of the scapula (T-C7) (n=6), and from the dyskinesis-free group (n=18), during the scapular dyskinesis testing (Type I: first; Type II; second and Type III: third line) performed in the scapular (left) and sagittal (right) planes.(¥)p=0.037; *p=0.0018 from non-paired t-test.

The clinical diagnosis and the 3D kinematic assessment were performed in a random sequence defined by lottery.

The clinical diagnosis was given by a physical therapy specialist in the area who had five years clinical experience working with disorders of the shoulder and upper extremity, and who was also a member of a research group on the shoulder joint complex. Prior to the study, this professional was trained to use the classification system proposed by Kibler et al.^5^ of the four types (type IV = normal) of dyskinesis, and provided with supplementary definitions. In addition to this material, pictures of the subjects with scapular dyskinesis were provided.

The clinical evaluation included a posterior and lateral view of each subject at rest with the arms along the trunk, and also during repeated arm elevation and lowering with the elbows extended, in the sagittal and scapular planes. The clinician diagnosed each scapula separately and asked each subject to hold the isometric position or to perform as many repetitions as deemed necessary for a conclusive diagnosis. The maximal number of repetions for test position 2 was 8.

The acquisition of the movements using the 3D kinematic sensors was obtained with the subjects' arms at rest and during elevation and lowering of the arm in the sagittal and scapular planes. The cycle of arm elevation and lowering in the sagittal (90º anterior to the frontal plane) and scapular planes (40º anterior to the frontal plane) were collected between the starting position and at the subject's maximum elevation point. The starting position was defined with the arms extended along the trunk, with palms of the hands facing the sagittal plane and thumbs pointing ahead^6^. Three sets of movements were collected in each plane, and the duration of each repetition was approximately four seconds or 90**º** per second. Visual feedback was not available for the participant during data collection. The data on the resting position of the scapula were extracted from records of the starting position and were collected after elevation and lowering movements of the arms.

The physical therapy specialist and those who participated in the data collection and processing of the data were instructed not to communicate with each other or to express their opinions on the diagnoses or results.

### Data analysis and statistics

*The Motion Monitor* (Innovative Sports Programs, Chicago, IL) was used to process the kinematic data. In this analysis, the variables that characterized each type of scapular dyskinesis were determined for comparison with the corresponding values obtained from the group classified as type IV (normal). Thus, the anterior tilt (medial-lateral axis) was used to define the diagnosis of type I; the medial rotation (longitudinal axis) of type II; and the superior translation of the scapula (frontal or scapular plane) of type III.

Data analyses were conducted on 51 scapulae, since kinematic data from one scapula was lost. Thus, the 26 subjects who participated in the study were divided into the following groups: type I (n=9, 18 scapulae), type II (n=5, 9 scapulae), type III (n=3, 6 scapulae) and type IV (n=9, 18 scapulae) based on the assessment following the fatigue protocol.

The values of the rotations of the scapulae, relative to the trunk around the medial-lateral and longitudinal axes, were extracted during the lowering phase (or eccentric phase) of arm elevation, according to the Euler angles sequence of rotation YX'Z'', as recommended by the ISB^14^. The values of scapular translation were extracted from the concentric phase of the arm elevation and corresponded to the linear distance between the trigonum spinae scapulae (root of the spine of the scapula) and the spinous process of the seventh cervical vertebra (T-C7).

Given that the clinical decision was determined by the observation of an excessive movement along the range of motion, the kinematic variables analyzed in this study corresponded to the difference between the maximum and the minimum value of the dysfunction. This corresponded to the normal pattern expected for the movement or the minimum value of the dysfunctional movement that occurred throughout the phase of movement assessed. The parameters of the interval used for analysis was established while considering the visual identification of changes in the direction of the curve for the kinematic variable of interest. In addition, the duration of the change in the direction recorded should be at least of 0.28 seconds(s). The longer the change in the movement direction, the easier it was for the clinician to indentify the dysfunction. In this case, 0.28 seconds, in addition to the peak value of the change in direction, was the minimum amount of time required to visually and cognitively diagnose dyskinesis^21^. Thus, even if the change in direction occurred more than once or if it was over the entire movement examined, such as the position of the scapula in medial rotation throughout the eccentric phase of the movement, the maximum value was included in the analysis.

The average values of the three measurements for each subject of the differences between maximum and minimum anterior-posterior tilt (type I), medial-lateral rotation (type II) and translation of the scapula (T-C7), were compared with the corresponding average values obtained from the subjects classified as normal (type IV). For this analysis, *Students'* unpaired *t* test was used with *Welch's* corrections applied where appropriate. *GraphPad Prism(r)* program (*GraphPad Software, Inc.,* La Jolla, CA) version 6.0 was used considering a statistical significance level of 0.05. The effect size of the comparisons was estimated by Cohen's *d*. The magnitude of the effect was interpreted as small when the value was 0.2; moderate at 0.5; and large when equal to or greater than 0.8^22^.

The levels of effort perceived were presented as a percentage of the change in the related categories, since the variable is nominal.

## Results

The average time determined for the muscle fatigue was 86 s (±37.2 s). For males, the average time was 114 s (±40.5 s) while for females, it was 67 s (±18.5 s). After the fatigue protocol, the subjects noted their level of perceived exertion, either between light and heavy (22.23%) or between intense and very intense (77.77%).

Only the comparison of the mean difference between maximum and minimum anterior-posterior tilt (type I) with the average values obtained from subjects classified as normal (type IV) was statistically significant, both in the scapular (p=0.0037, Cohen's *d*=0.778) and sagittal planes (p=0.0018, Cohen's *d*=1.128), indicanting a greater anterior tilt of the scapula for those with type I diagnoses (Figure 2). For the non-significant comparisons, the magnitude of the effects was small, with Cohen's *d* values ranging from 0.06 to 0.493.

## Discussion

The objective of this study was to compare the kinematic data indicative of scapulothoracic rhythm dysfunction between subjects with and without a diagnosis of scapular dyskinesis, using the classification proposed by Kibler et al.^5^. Our results showed that, independent of the plane of motion in which the test was performed, the difference between the maximum and minimum anterior tilt of the scapula was greater in the group of subjects identified with dyskinesis type I, characterized by the dorsal prominent of the inferior medial border of the scapula, compared to the values of the group diagnosed as normal.

Regardless of the plane in which arm elevation and lowering was performed, the variables indicative of dyskinesis, characterized by prominent of the medial border of the scapula or superior translation of the scapula, showed no significant differences when compared to the normal group. Although the results may indicate a lack of validity in the diagnosis of dyskinesis type II and III conducted by the physical therapy specialist, it is important to highlight that the sample size of these groups does not support this conclusion.

A previous study has shown a standard deviation for the scapulothoracic motion to be 10º. Considering an effect size of 5% for the differences between the variables, a power of 80% and an alpha of 5%, the estimated sample size calculated was 12^23^. Thus, the group diagnosed with dyskinesis type II had 75% of the minimum sample size, while the group with type III had only 42%. The interpretation of the Cohen's d, for the magnitude of the effect size of the differences found in the anterior tilt of the scapula in the scapular and sagittal planes, indicated moderate and large effects, respectively. The low prevalence of asymptomatic subjects types II and II in the sample did not allow us to reach magnitudes of appropriate effect to validate the clinical observation of these dysfunctions.

Only one study^6^ attempted to validate the criteria of dyskinesis proposed by Kibler et al.^5^, albeit with little success, due to the high prevalence of asymmetry in both groups with and without a diagnosis of dyskinesia. Uhl et al.^6^ compared the 3D kinematics of volunteers assessed by clinicians using Kibler et al.^5^'s four type classification and the method "YES" (either type I, II and III) and "NO" (type IV), with reference values reported in a pilot study. These reference values were obtained from the resting position of the scapula of eight healthy subjects with no clinical diagnosis of dyskinesis, assessed by the clinicians. These values were calculated as the difference between the right and left medial-lateral rotation, and from the anterior-posterior tilt and vertical translation of the spine of the scapula with respect the spinous process of the twelfth thoracic vertebra (T12). In the present study, we proposed the new variable maximum - minimum to analyze the displacement in the position of the scapula. This variable eliminated using data from both scapulae, as bilateral comparison in static test^24^ was previously questioned^4^ in the literature, since the dysfunction could affect the scapula position bilaterally. Besides the individual evaluation for each scapula, our 3D variable favors key components of the clinical diagnosis. Data arises from scapular in motion and adds the judgment of excessive motion (maximum value) and the expected or minor dysfunctional motion (minimum value) within a time compatible to the cognitive interpretation^21^ of the examiner.

In the present study, a fatigue protocol was performed by the subjects prior to the dynamic examination of dyskinesis. Previous studies have shown that the effect of muscle fatigue on the kinematics and postural control of the lower limb worsens the muscular dysfunction^18^ ^,^ ^19^. Similarly, to facilitate the identification of the dysfunction, repetitions of arm elevation with submaximal loads and in the *push-up plus* position were performed until fatigue was apparent, since the coordination of the movement and much of the stability of the scapula on the thorax is given by the dynamic participation of the axioscapular and scapulohumeral muscles^8^. It is also necessary to know whether symptomatic volunteers will be able to perform the fatigue protocol proposed, and if it is more efficient than the use of loads along the arc^7^ of movement, since the fatigue protocol could be a condition of increased demand^24^ compared to the demand provided by using loads that vary during the movement.

Therefore, the limitation of the study was the inability to confirm the clinical observation diagnosis of the four types of scapular dyskinesis due to the low prevalence of types II and III in the sample evaluated. Future studies should determine the validity of these categories in order to make the clinical observation test acceptable for the diagnosis of scapulothoracic rhythm dysfunction. It should also include asymptomatic subjects and the estimates of sensitivity, specificity and accuracy.

## Conclusion

According to this study, the clinical observational assessment of the scapulothoracic rhythm was considered appropriate only for the diagnosis of dyskinesis type I, characterized by excessive dorsal projection of the inferior medial border of the scapula. Considering the low prevalence and sample size of types II and III, further studies are needed to validate the clinical observation method as appropriate for the diagnosis of scapular dyskinesis.
